# Retained Primitive Reflexes and Potential for Intervention in Autistic Spectrum Disorders

**DOI:** 10.3389/fneur.2022.922322

**Published:** 2022-07-07

**Authors:** Robert Melillo, Gerry Leisman, Calixto Machado, Yanin Machado-Ferrer, Mauricio Chinchilla-Acosta, Shanine Kamgang, Ty Melillo, Eli Carmeli

**Affiliations:** ^1^Movement and Cognition Laboratory, Department of Physical Therapy, University of Haifa, Haifa, Israel; ^2^Department of Neurology, University of the Medical Sciences of Havana, Havana, Cuba; ^3^Department of Clinical Neurophysiology, Institute for Neurology and Neurosurgery, Havana, Cuba; ^4^Department of Neuroscience, Carleton University, Ottawa, ON, Canada; ^5^Northeast College of the Health Sciences, Seneca Falls, New York, NY, United States

**Keywords:** top-down processing, bottom-up processing, neuronal synchrony, maturational delay, autism spectrum disorders, retained primitive reflexes

## Abstract

We provide evidence to support the contention that many aspects of Autistic Spectrum Disorder (ASD) are related to interregional brain functional disconnectivity associated with maturational delays in the development of brain networks. We think a delay in brain maturation in some networks may result in an increase in cortical maturation and development in other networks, leading to a developmental asynchrony and an unevenness of functional skills and symptoms. The paper supports the close relationship between retained primitive reflexes and cognitive and motor function in general and in ASD in particular provided to indicate that the inhibition of RPRs can effect positive change in ASD.

## Introduction

### What Are Retained Primitive Reflexes and What Is the Controversy?

The term “primitive reflex” was first used by Buckley (1). Primitive or infantile reflexes are sensory/motor reflexes that are present at birth. Most of these reflexes are present in the womb (2), and one of their functions is to help the child “birth itself.” The primary function of primitive reflexes is to allow the infant to move and react to their environment leading to the maturation of the motor system (3, 4). Children need to move, feed, protect, and orient to engage their senses and muscles and create sensory and motor feedback that will activate genes allowing the brain to be built from the bottom up (3–5). The control of these reflexes arises from multiple brainstem regions. The lower reflexes in the medulla are thought to be active first, followed by reflex control associated with the pons and mesencephalon (6–10).

Nevertheless, if there is a delay or disruption to this bottom-up projection known as “bottom-up interference,” then the later, more advanced areas of the brain may be delayed in development. This could then delay or prevent the top-down maturational processes that ultimately inhibit these reflexes (11–13). Babinski also noted that not only the response delay of the downward toes in the plantar reflex but also the asymmetry of this response had clinical significance. Asymmetry of the Babinski sign is significant and may relate to a functional maturational dysfunction of the corticospinal tract (14–16).

Several authors have emphasized that frontal lobe development eventually leads to top-down control and inhibition of primitive reflexes. If there is degeneration or damage to the frontal lobe or corticospinal tract later in life, these reflexes can return. They are considered to be frontal release signs (17–20).

**Figure 1:**
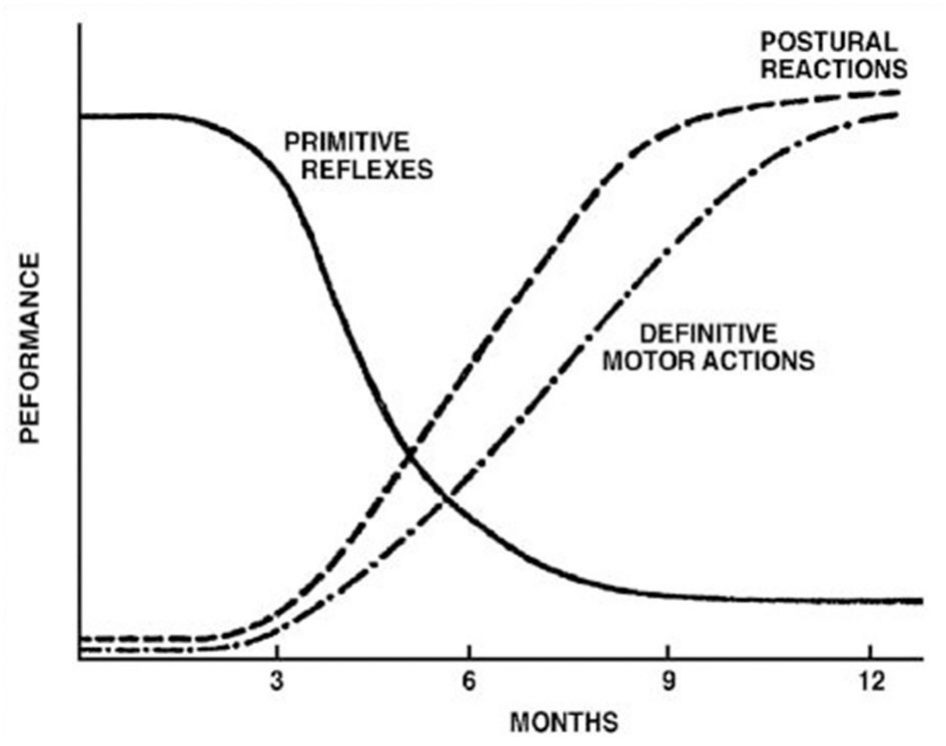
Development of postural reflexes. The diminishing of primitive reflexes and the growing importance of postural reactions indicate the development of requisite conditions for the development of the purposeful movement. The collective time course of primitive reflexes under normal circumstances is compared with the time course of the maintenance of static deep tendon reflexes and postural reactions. Reproduced with permission Pedroso FS. Reflexes. In: Haith MM, Benson JB, editors. *Encyclopedia of Infant and Early Childhood Development*. San Diego, CA: Academic Press (2008). p. 11–23.

**Figure 2:**
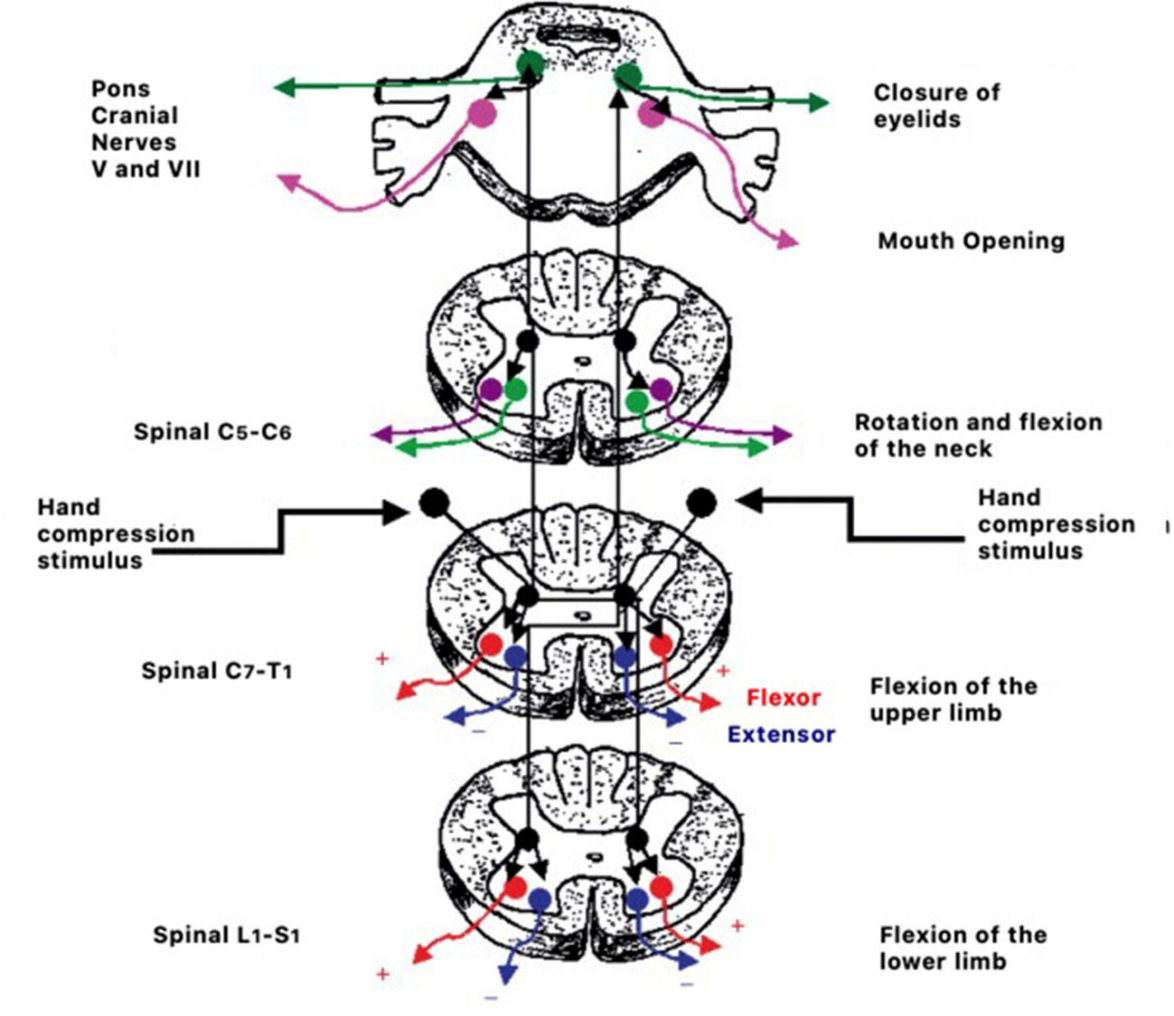
Example of the circuitry of reflex responses to hand compression stimulation.

The controversy surrounding RPRs is not whether they exist or not. Although not typically part of the current pediatric examination, primitive reflex testing was previously included as part of a routine pediatric neurology examination. They are, however, a well-accepted part of the evaluation of effective child development (3, 7, 21–23), whose normal temporal trajectory is reported in Figures 1, 2. The controversy surrounds the inhibition of these reflexes. In mainstream pediatrics, it is assumed that primitive reflexes are completely inhibited by the end of the infant's first year postpartum. However, many studies have indicated that in a certain percentage of the population, primitive reflexes are not inhibited in the first year of life and persist into middle childhood and even into adulthood (24, 25).

It has also been documented that in children, adolescents, and adults with RPRs, a neurobehavioral disorder or “learning disability” coexists (4, 26, 27). Individuals with ADHD, (25) autism, (28) Tourette's, (29) dyslexia (30), or other neurobehavioral disorders, frequently demonstrate RPRs that are thought to be related to maturational delay in the nervous system (3, 31–34).

Movement allows us to interact with the environment in more sophisticated ways and can help improve our chances of survivability. Interacting with the environment purposefully and beneficially requires the development of sensory organs that supply the individual with information about our location, potential danger, and satisfaction, allowing one to negotiate the surroundings and develop a sensory-motor map of the world that we can use for prediction and goal-directed adaptive behavior (35, 36).

Organisms developed brains because they moved, and as they moved, they interacted with their environment in increasingly complex ways, leading to the development of a more complex brain (37, 38). Bipedalism is the most complex of movement strategies that have evolved in any organism, and it is, for the most part, unique to hominids. The relatively large brains of humans support the distinctive erect bodily position and cognitive abilities driven by bipedalism (39). Upright bipedalism permits less flexibility of the human pelvis's anatomy and size and structure in the human pelvis compared with quadrupeds (40). Relatively large-headed infants are born to mothers with relatively small birth canals. The infant's skull and brain cannot be fully developed at birth for the mother and neonate to survive the birth process unscathed (41, 42).

To stimulate the growth and development of the brain, an infant needs to move and interact with its environment (43–45). Movement can be impeded by a brain and nervous system that is not sufficiently mature at birth. What makes some volitional movement possible are the infantile primitive reflexes already intact at birth that allow for reflexogenic movement and interaction with the environment in fundamental ways that help increase the chances of survival. These reflexes appear prenatally and are thought to aid in the birth process. Most of these reflexes are present at birth and then become inhibited within the first few months, with the longest (the plantar reflex) remaining until the end of the first year postnatally. These reflexes allow for basic reflexogenic movement contributing to early motor milestones such as rolling over, creeping, crawling, grasping, sucking, and eventually crawling and walking (43, 44). Postural reflexes that allow for more sophisticated individualized movements are replaced with voluntary movement in most cases. Primitive reflexes allow for basic movements, which allow for simple interaction with the environment and form the basis of the early movement as well as in the stimulation of sensory organs and receptors. This increase in sensory feedback and stimulation is thought to result in the expression of genes related to protein synthesis and the building of functional connections (46). The stimulation of glial cell proliferation increases the size and connectivity of neurons (47). As neurons grow in size, density, and connectivity, they will eventually inhibit, through propriospinal projections, lower or more primitive areas of the brain. They will stimulate the growth and activation of higher, more sophisticated regions of the brainstem and neocortex. While primitive reflexes eventually become inhibited or integrated, they are never entirely eliminated (48). Ultimately, all reflexes seem to come under the control of the frontal lobe (17, 18, 49–51).

In individuals with frontal lobe damage, dysfunction, or degeneration, the reappearance of primitive reflexes known as frontal release signs is oftentimes manifested (17, 18, 34, 49–56).

Upper motor lesions also do not infrequently result in the reappearance of primitive reflexes such as the Babinski reflex or plantar reflex (57). This is thought to be associated with the loss of the descending inhibitory connections from the cortical spinal tract, which reflects the maturation and growth of the frontal lobe and the sensory-motor cortex.

It has also been noted (58) that the presence of RPRs is a common feature of children with Autism Spectrum Disorder (ASD) (28, 58–60). In most of these disorders, there is no visible damage, injury, lesion, or degeneration as a basis for hypothesizing that the RPRs reflect a maturational delay of brain areas that would typically inhibit these reflexes, especially those in feedback with the frontal lobes (61–63).

The absence or reduction of environmental influences that would generally promote growth and development, and neuroplasticity within higher brain regions, would typically lead to the inhibition of primitive reflexes and the expression of postural reflexes (3, 64) according to the timeline represented in Figure 1 and in Table 1. The persistence of these primitive reflexes can reflect a maturational lag. The RPRs, especially with asymmetric persistence, will reflect not only a maturational delay of the brain but may also indicate, depending on the timing, abnormal asymmetrical development of the hemispheres (64, 65). In children with cerebral palsy, an injury on one side of the brain can lead to asymmetric retention or lack of development of PRs (28, 66, 67).

**Table 1:** Primitive reflex development and integration timetable.

	***In utero*** **(in months)**									**First year of life (in months)**														
	**1**	**2**	**3**	**4**	**5**	**6**	**7**	**8**	**9**	**1**	**2**	**3**	**4**	**5**	**6**	**7**	**8**	**9**	**10**	**11**	**12**	**13–24**	**25–26**	
TLR-flexion																								**Lifelong Reflexes**
TLR-extension																								
ATNR																								
STNR																								
Grasp																								
Palmar/Babkin																								
Plantar																								
Babinski																								
Dear paralysis																								
Moro																								
Spinal galant																								
Head righting																								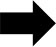
Spinal perez																								
Landau																								
Amphibian																								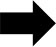
Crossed extensor																								
Rooting																								
Sucking																								

According to our current understanding, the prevalence of RPRs is considered variable, and there is disagreement about the pathological significance of these reflexes in both aging and child development. However, evidence from large data sets indicates a significant relationship between RPRs, maturation, and cognitive function (3, 25), and the description of these reflexes is presented in Table 1.

### Retained Primitive Reflexes vs. Returned Primitive Reflexes

The relationship between cognitive deficits and RPRs has been controversial. Some authors consider these reflexes predictive of diffuse cerebral dysfunction as these signs are significantly correlated with cognitive deficits in a wide age range of individuals (68–73). It is therefore important to differentiate “*retained primitive reflexes*” from “*returned primitive reflex*” (RtPR). Recently, RtPRs have been described in dementia and Parkinson's Disease (74–78). While primitive reflexes are considered adaptive responses that are present in the neonate and disappear or are inhibited as the brain matures, RtPRs can reappear in childhood, adolescence, and adulthood (78) and when they do so, they are reportedly invariably associated with cognitive effects (74, 78). Some authors consider that RtPRs (in particular the Babinski and grasp reflex) are indicative of diffuse cerebral dysfunction as there exists a significant correlation between these signs and cognitive dysfunction in a wide age range of individuals (69–73). Regarding RtPRs, some authors reported that in individuals with Alzheimer's disease, no relation existed between cerebral atrophy based on psychometric testing (e.g., Wechsler Memory Scale) or CT-scan and grasp, snout, or glabellar reflexes.

Retained primitive reflexes indicate cortico-subcortical neuronal network impairment or possibly neuronal developmental delay. Some authors have stated that RPRs are evidenced in neurotypical populations. The palomental reflex, for example, was found in 6–27% of individuals aged between 20 and 50 years, and 28–60% of those above 60 years (64), snouting in 13% of individuals between 40 and 57 years (64); 22–33% of those above 60 years of age (64), and the sucking reflex, which some authors associate with “frontal lobe disease” (79), and Tarawneh and Galvin (80) had noted that in neurotypical individuals between 73 and 93 years of age, the palmomental reflex was evidenced in six percent them.

The Babinski sign (i.e., plantar response) and grasp reflex are two reflexes that are clinically accepted as indicators of central nervous system disease or disorder. Some of the arguments may be accounted for by differences in opinion and interpretation of the reflexes, which can vary significantly from clinician to clinician (4, 26, 81–83).

Another study examined the relationship between cognitive functioning and RPRs in individuals with dementia and without in order to determine the most predictive elements of cognitive testing or the neurological examination for brain dysfunction (84). Using the Cognitive Abilities Screening Instrument-Short Form (CASI-S), Gellis (85) concluded that in those with dementia, individuals with the highest primitive reflex (PR) scores tended to be associated with the lowermost cognitive scores and, in particular, to SPECT scan configurations. Therefore, these researchers concluded that the existence of numerous PRs and cognitive scores could be effective in predicting diffuse cerebral dysfunction. In particular, the presence of the Babinski and grasp responses, or the combination of the snout, suck, paratonia, and palmomental reflexes, are effective indicators of diffuse brain dysfunction, in particular when RtPRs are evidenced and complemented by deficits in cognitive testing scores.

The presence of multiple primitive reflexes is an indicator of diffuse brain dysfunction in elderly populations. Their persistence and presence in children and adolescents may indicate diffuse cortical maturational delay and correlate with cognitive and executive developmental absence or delay. If developmental milestones are not appropriately achieved, we hypothesize that synchronicity, optimization, the efficiency of behavioral-environmental interaction, coordination of movement, and synchronization of the overlapping brain will all be affected (25, 27, 43, 44, 86).

## Primitive Reflexes, Neuronal Synchrony in Cortical Development in ASD, ADHD, and Other Neurobehavioral Disorders

### Maturational Delays and Lateralization

One of the unique features of the human brain is its degree of lateralization or asymmetry. Humans have the most asymmetrical and lateralized brains of any species. This is thought to be another factor that leads to the significant differences in intelligence between humans and other species. A more lateralized brain allows for the development of a greater variety of centers that can individually process and control numerous functions, combining these individual centers into various networks leading to the unique cognitive abilities shared by humans. This lateralization develops with increasing age, brain, and nervous system development (87–93).

A small child does not have the same degree of lateral asymmetry as an adult. Laterality is a product of the maturity of the brain and especially of the neocortex and the frontal lobes (43, 89). The development of laterality and asymmetric control of functions increases the cognitive potential and requires greater coordination and synchronization of cortical networks (94, 95). For various functions to bind together, all associated brain regions that control complex functions and their networks must be activated simultaneously. This coordination is a byproduct of maturity (4, 21, 43).

As the brain grows and as neurons become interconnected, the speed and coordination of inter- and intra-hemispheric cortical networks increases, allowing for synchronization and integration of a significantly greater number of functions. The two hemispheres of the brain do not develop simultaneously; the right hemisphere is thought to develop more rapidly and earlier than the left, with the most significant development being prenatal and for the first 2–3 years of life (96, 97). Then the left hemisphere is significantly more greatly emphasized in development for the next 2–3 years of life. Once the differences and advantages are established during the first 6 years of life, this forms the basis of hemispheric specialization and lateralization that will increase throughout development (87, 90–93, 97).

### Asymmetric Development Can Lead to Underconnectivity, Desynchronization, and Functional Disconnection

Where neural connections (anatomic and functional) are not adequately developed in infancy and early childhood, asynchronicity, the inefficiency of behavioral-environmental interaction, coordination of movement, and synchronization of brain networks may be evidenced (40, 98–104).

A global immaturity of the function of cortical networks in childhood can be associated with a reduction in motor activity (3), spatial exploration (105), experience-dependent plasticity (106), RPRs and delayed postural reflexes (107, 108). A more specific dysfunctionality would be expected if there was asymmetric development of RPRs. If there existed unilateral RPRs and, in particular, unilateral delay of postural reflexes, we would expect an asymmetric maturity and growth of the brain since this would be associated with an asymmetry of tone, in turn altering sensory and muscle feedback, potentially impairing the main driving factors to brain development (109).

Futagi et al. examined the relationship between plantar grasp response asymmetry during infancy and neurological outcome. They, during a follow-up period of between 2.8 and 11.9 years, reviewed the neurologic consequences of 61 children having demonstrated, during infancy, asymmetric plantar grasp responses. All children demonstrated neurological signs or perinatal risk factors during infancy. Futagi and colleagues reported intellectual disabilities in three, borderline intelligence in nine individuals, cerebral palsy in 38, delayed motor development in six, and neurotypical function in five. The majority exhibited a relationship between the side of the retained plantar grasp response, the side of the motor function deficits, and the side of the abnormal CT results (6). These findings were supported in a systematic review by Hamer and Hadders-Algra (110).

The asymmetry that Futagi et al. (1995) observed in the plantar grasp response strongly suggested brain dysfunction (6, 111, 112). Their studies showed an association between the persistence of motor abnormalities related to the same body side and the asymmetric development of primitive reflexes (113). The plantar response is one of the reflexes most related to brain dysfunction, whether due to injury or functional developmental delays (108, 114). This type of asymmetrical development of the developing brain has been commonly noted in almost all neurobehavioral disorders, especially ASD (115, 116). Along with anatomical asymmetries, there have also been functional asymmetries noted with a characteristic “unevenness” of skills observable in all of these disorders, to varying degrees (25).

A significant feature of those with ASD is the “unevenness” of cognitive function (117). We have proposed (43) that the diverse aberrant behaviors noted in ASD and in other neurobehavioral disorders can be understood better by viewing ASD in the context of functional brain disconnectivity, of the kind that has been noted in minimally conscious states (118, 119) and even in sleep (120), or as reported in people with dyslexia (121, 122). Functional asymmetry within widespread cortical networks could decrease temporal coherence in certain functional networks and enhance temporal coherence in others (123). Recent research has suggested that increased functional ability or intelligence is related to augmented activity in specific networks (124). It is also possible that an increase in the complexity and integration of functional networks may be related to increased temporal coherence that may impart a selective advantage in particular regions of the brain (125, 126). This could explain how certain talents and abilities seem to be inherited and run in families (127).

As optimized brain function implies more efficient neural processing than non-optimized, one might expect optimized execution of motor tasks to be related to greater degrees of activity. However, the converse appears to be the case in the cerebral cortex where increased task effectiveness has been reported that has included: figural, numeric, and spatial reasoning (128) and verbal ability (129) is associated with reduced energy consumption in various cortical regions. This phenomenon has additionally been examined electrophysiologically. When examining resting-state activity [event-related desynchronization (ERD)] during cognitive tasks, there is a reported decrease in background power (7.5–12.5 Hz) decreases which has been reported to be related to the activity recorded in those with higher scores on IQ tests (130, 131), or with significantly greater performance after practice, that in turn is related to a more effective cognitive processing strategy (132, 133). Yet, the issues should include not only the expenditure of energy but also the nature of the functional connectivities between brain regions (94). Smaller regions of activity have been consistently evidenced in brain areas in those with ASD. These diminished areas of activity appear to be developmentally delayed brain regions as opposed to being reflective of pathological processes or damage (94, 95).

Individuals with ASD and other neurobehavioral disorders have also evidenced a reduction of interregional brain connectivity (25, 27, 43, 44, 94, 95, 102, 134–139). The corpus callosum appears to be the brain area associated with the reduced cortical connectivity found in individuals with ASD (140). This implies that the most frequently evidenced functional disconnectivity observed in childhood involves hemispheric interaction. This is a notable reported characteristic difference between ASD and normally developing toddlers (141).

We think that reduced inter-hemisphere coherence is associated with a reduction in the several sensory, motor, and cognitive functions coordinated by the ipsilateral brain hemisphere, and the higher proficiencies are sometimes related to enhanced within-hemisphere coherence (43, 94, 95). We have also described diminished coherence and connectivity in longer inter-hemispheric connections with augmented coherence and connectivity with shorter intra-hemispheric connections (94, 95) that we have hypothesized to be associated with enhanced performance abilities such as those that have been observed in forms of savantism (142).

## Retained Primitive Reflexes, Motor Function, and Neurobehavioral Disorders

Retained primitive reflexes have been noted in several neurobehavioral disorders, including ADHD and ASD and are understood to be associated with or absent or delayed developmental milestones in these (25, 27, 28, 44, 143–148). RPRs have been reportedly associated with the presence of clumsiness (25, 27, 43, 149, 150) incoordination (149), awkward posture (151), gait (152–154), and other motor disturbances (25, 155, 156). Most neurobehavioral disorders seem to be associated with motor incoordination and cognitive dysfunction (25, 27, 43, 44, 157, 158).

Teitelbaum et al. (159) theorized that in infants with movement disturbances, reflexes may have “gone astray” and may be early markers of ASD. They observed that some infants demonstrated RPRs that continued far beyond long infancy in the children they examined, whereas other primitive reflexes first appeared in infancy significantly later than though ought. The asymmetric tonic neck reflex, they thought, might be retained in ASD. The verticalization of the head as a consequence of body tilt, was reportedly absent in a subgroup of “autistic-to-be” infants, according to Teitelbaum and colleagues. They suggested that these reflexes might serve as a marker for ASD, and pediatricians could use them to screen for neurological dysfunction (160). In their earlier work, Teitelbaum et al. (161) showed that infants with a tendency to ASD demonstrated a distinctive constellation of disturbances in patterns of movement as early as 4–6 months of age, measured by Teitelbaum and colleagues in conjunction with laser disc still-frame analysis. Eshkol and Wachman (162) had earlier reported similar findings.

The Galant and Moro reflexes are among the most critical postnatal primitive reflexes that diminish later in development. At the same time, there exists no definitive evidence that these reflexes play a role in ADHD. Konicova and Bob studied school-aged ADHD children between 8–11 years who demonstrated Galant and Moro RPRs compared to an age-matched control group (72, 163). They found that ADHD children demonstrated a significantly greater occurrence of Moro and Galant reflexes than did the control group, indicating that ADHD symptoms may compensate for an immature brain.

Callcott (164) reported that children's learning difficulties relate to reduced movement proficiency, including school-readiness. Calcott investigated the prevalence and severity of the Asymmetrical Tonic Neck Reflex (ATNR) and studied the proficiency of movement in preprimary-aged Western Australian indigenous children. She found that 65% of those tested demonstrated moderate to high ATNR levels that were significantly related to academic achievement (164).

The findings of the Millennium Cohort Study in the United Kingdom (165) supported a relationship between the delayed achievements of motor milestones at 9 months of age and significantly lower cognitive development at age five (165). The Australian Early Development Index reported that nearly a fourth of school-aged are “at risk” in their physical and cognitive development (166). Williams and Holley (167) offered support for these findings linking motor development and cognition by addressing the influence that infant motor experiences in infancy and early childhood may have on higher-level cognitive abilities required for academic achievement in school. As we have already noted, motor function and gesture development typically require the effective inhibition of mouth and hand-related primitive reflexes (167). ASD children not infrequently demonstrate difficulty in executing skilled movements and possess as well as exhibit a poor gesture repertoire (168).

Chinello et al. (144) examined the association between three RPRs, motor behavior, and parental autistic-like traits, in infants aged between 12 and 17 months of age. Independent of age, RPRs were associated with infants' deficient motor skills and were highly correlated with parental autistic-like characteristics.

Numerous authors have reported on an association between clumsiness and incoordination, particularly in gait and posture, and ASD, ADHD, and other neurobehavioral disorders of childhood (163, 169, 170). The kind of gait and motor dysfunction has been mainly thought to possess either basal ganglia or cerebellar origins (25, 27, 40, 43, 171). Developmental Coordination Disorder (DCD), or more simply put, motor incoordination or “clumsiness,” is also usually of the same type, primarily involving the muscles that control gait and posture or gross motor activity (172, 173).

Sometimes, we observe that fine motor coordination can also be affected (21, 174, 175). Several authors have noted both differences and similarities between ASD and DCD. DCD individuals demonstrated greater fine and gross motor coordination, theory of mind, and emotional perception than did the ASD individuals, but evidenced comparable difficulties with response inhibition. These authors observed that based on symptom severity, children with ASD who were measured to be “more able” did not diverge on any measured skills from DCD children, in contradistinction to children classified as “less able.” The authors wondered whether DCD and ASD vary more in the range of symptom severity than in a singular behavioral domain (174).

Similar comorbidities have been found in children with ADHD (72, 163, 176–180) and those with developmental dyslexia (4, 181–186).

Sumner et al. (187) also found numerous overlapping features in verbal expression, speech, gaze, and face-processing, expression, in ASD and DCD individuals. These findings suggest that children with DCD may also demonstrate difficulties in processing social information. However, when examined with measures of socialization, the DCD individuals scored at an intermediate level in two other socialization measures. The authors concluded that socialization in DCD may not be as manifest as in individuals with ASD (187).

The greatest similarity between ASD and DCD in Sumner and colleagues' review was a paucity of significant effects of cognitive intervention. Concerning treatment, no significant improvement effects were noted in both DCD and ASD groups of individuals (174) or in IQ (188). The ability to train and improve in various domains, especially cognitive, is similar for both conditions. Additionally, no significant disparities were found in a qualitative study examining transitions from primary to secondary school, possibly because the main variables of the study's interest concerned the children's motor behaviors. On the other hand, cognitive intervention has been reported to be effective in reducing symptoms of ADHD (189).

It might seem somewhat confusing initially to observe that fine motor skills seem to be disrupted at almost equal levels as a gross motor. The literature supports the notion that manual dexterity is less effective for high functioning ASD individuals, but only for the non-dominant hand. This suggests a lateralized difference (190, 191). This would show that although fine motor coordinative skill is decreased in those with ASD and fine motor skill is primarily decreased in the left hand, associated with right hemisphere function. This is consistent with a deficit in effective coherence between the right and left hemispheres. Perhaps a parallel situation exists in ADHD individuals and in individuals with other neurobehavioral disorders (192–196).

Variations in the manifestations of ASD and DCD may be associated with differences in the maturation of asymmetries as a consequence of different maturational rates of the left and right hemispheres (197–199). Asymmetric RPRs may also be an early marker of developmental brain immaturity. This aberrant configuration of hemispheric asymmetry may be related to underconnectivity and desynchronization, and eventually to functional disconnectivity between lower brain regions and the neocortex (197–200).

### Retained Primitive Reflexes in ASD

Autistic Spectrum Disorder is a neurobehavioral disorder identifiable by dysfunction of communication, behavioral flexibility, eye contact, and social interaction as well as deficits in language, and executive function (201–204). Although there is a consensus about the symptoms that comprise ASD, there exist controversies regarding the precise definitions of ASD and the boundaries between manifestations of related disorders. Researchers have increasingly recognized that motor ability can have a significant effect on other developmental functions, such as language and social cognition (28, 205–209).

Retained primitive reflexes can disturb the natural course of development and create difficulties in social and educational functions in children (21, 210) as well as impact psychomotor development (211). Mature responses in a child's psychomotor behavior can only occur if the central nervous system has reached the appropriate level of maturity (21, 210, 212). The process consists of the transition from brainstem reflex response represented in Figure 2 to cortically controlled responses (213).

It has been argued that independent of a child's age, RPRs are significantly related to an infant's ability to interact with objects (i.e., agency) (4, 21, 144, 156, 210) as well as with others (i.e., copying gestures) (22), meaning that high scores in the assessment of primitive reflexes, is associated with an increased likelihood of RPRs, which, in turn, are also associated with low scores in motor responsivity, independent of the age of the infant.

As previously indicated, children with ASD demonstrate impediments in the performance of skilled movements and gestures. Numerous investigators have noted that delays in the maturation of motor function during the early years of development foretell the primary dysfunctions characteristic of individuals with ASD (214–216).

This hypothesis has been examined in the infant siblings of ASD children, who purportedly have an increased probability of developing ASD. In longitudinal studies of 3- to 6-month-old infants' motor development of high-risk (HR) infants, over 70% of infants with motor delay later demonstrated communication impairment. Motor development is associated with a normally automatic progression in which infant maturation inhibits more primitive motor responses (217–219).

Assessing RPRs in autism is essential for at least multiple purposes. Firstly, RPRs may be an encouraging early sign of ASD that, along with the early signs of difficulty in eye contact, attentional deficits, as well as other elements, might assist in characterizing the developmental trajectory of the wider ASD phenotype during infancy. Consistent with this thinking, it has been stated that slight disparities in initial periods of the brain's development (e.g., the persistence of primitive reflexes) can produce an adverse progressive effect not just on motor skills that develop later but also on a range of other behaviors (i.e., communicative and social behaviors as well as in object exploration) (144, 220, 221). Secondly, the ability to identify motor abnormalities early in life might also be encouraging for the differential diagnosis of ASD. The proportion of children with ASD and concomitantly with developmental motor and coordination dysfunction varies widely. The variability in these deficits in ASD is likely a result of the heterogeneity of ASD. That heterogeneity, on the other hand, allows us a unique opportunity to classify subtypes of ASD (101, 194, 195).

Retained primitive reflexes have the ability to disturb the normal maturation processes decreasing the ability of the brain to effectively process sensory information. RPRs then that are still present (beyond the average age of 12 months postpartum) can impede the subsequent development and maturation as well as serve as a potential biomarker of neurological dysfunction (144).

### Do RPRs Indicate a Dysfunctional Neurological System in ASD?

In supporting the cognitive effects of primitive reflexes and cognitive function, some authors consider the palmomental reflex (PMR) as being related to dementia. The PMR is a polysynaptic reflex that can be evoked by nociceptive stimulation of the thenar eminence, resulting in an ipsilateral involuntary mentalis muscle contraction (19). The extant PMR is found in infants up to ~12 months of age and then wanes and disappears, largely due to the frontal lobe maturation (19, 222). Consequentially, its recurrence in aged individuals with the pathology of the frontal lobe is thought of as a “cortical release” or “frontal lobe” sign, with a presumption of a lack of frontal inhibition on subcortical motor networks (223–225).

Anatomic (AC) and functional connectivity (FC) studies of linking the PMR with dysfunction of interrelating loops connecting the thalamus and basal ganglia with the motor, premotor, and prefrontal cortices, are consistent with our hypothesis (95, 190, 226–229).

Neuroimaging studies of individuals with ASD have also detected brain areas with atypical lateralization of motor function, with the capacity to detect subtle neuroendocrine phenotypes. Most studies agree that ASD individuals demonstrate an amplified rightward asymmetry that incorporates cerebral cortex volume, corpus callosum, premotor cortex, the sensorimotor resting network, and the inferior parietal lobule (190, 227, 228, 230, 231).

Floris et al. (190), in studying intra-hemispheric connectivity in ASD, demonstrated that high-functioning ASD children aged between 8 and 12 years demonstrated strong rightward lateralization in their motor circuitry's connectivity which was found to be necessary for effective motor responsivity. Notably, motor connectivity's rightward lateralization relates to effective motor response (e.g., gait and balance, as well as any timed sequential or movements) (190, 231).

Machado et al. (94) reported that qEEG changes in coherence and spectral analysis could be associated with a visual-auditory sensory integration impairment, which in ASD children is lateralized to the right hemisphere (94, 232, 233). Hence, several authors have affirmed that RPRs reflect anatomic and functional connectivity abnormalities in brain networks (190, 227, 228, 230, 231).

### Do RPRs Reflect Motor Impairment in ASD?

Several authors have suggested that RPRs correlate with motor function independent of the age of the infant, and significantly more so among infants whose parents demonstrated subclinical autistic characteristics. Hence, the RPRs might modify the developmental trajectory of the infant's motor function, and as a result, their assessment could serve as an early marker of atypical development (144, 156).

There exists a consensus that besides the principal features of ASD, delays, and abnormalities in motor development are key components of ASD regardless of multiple etiology and subtypes of the condition (25, 27, 44, 144, 156).

The anomalous frontostriatal cortex in AD individuals with RtPRs variously affects the motor pathways that suppress primitive reflexes. This notion is coherent with findings that ASD impacts various cognitive functions in the same way that dementia is related to dysfunction of the frontal lobes and/or basal ganglia (i.e., frontotemporal dementia or Parkinson's disease dementia). These findings in dementia have suggested functional and anatomic connectivity impairment in ASD and other neurodevelopmental disorders (19, 224).

## Retained Primitive Reflexes Can be Biomarkers and a Target for Treatment

Retained primitive reflexes may be one of the earliest markers of abnormal or delayed cortical maturation and by extension, of ASD and other neurobehavioral disorders (231, 234). The rooting and sucking reflexes and many other primitive reflexes are present at birth (43). The inability of an infant to attach to its mother and breastfeed, often seen in children with developmental delays and delays or asymmetry of rolling over at 3–5 months of age, may be the early indicators of ASD (3, 77, 235). Therapists have recommended exercises that stimulate or reproduce primitive reflexes to remediate various neurobehavioral disorders (26, 149, 236).

Methods that have indicated some promise in the treatment of various neurodevelopmental disorders including ASD are ostensibly founded on the theory that attributes the difficulties to RPRs that affect the child's normal growth and development as well as academic and cognitive skills (25, 237–239).

Although limited, some studies indicate a neurodevelopmental basis for a range of difficulties associated with maturation and motor development that manifest in cognitive and social difficulties. The collective research in this area demonstrates that the existence of RPRs has implications for skills such as balance and coordination as well as learning and cognition. The work of Goddard Blythe (240–243) has concentrated on children between 7 and 9 years of age and supports the case for early interventions to improve and develop coordination and balance, especially when such neurological dysfunction may be contributing to cognitive and motor delays or effects. Brown's (244) intervention study appears to support this line of research as does Melillo et al. (25). She found that with children between 4 and 5 years of age, practicing particular movements facilitated their performance of the fine motor activity and academic performance by inhibiting RPRs. Similar findings were reported by McPhillips and Mulhern [(239), cited in (245), p. 69] who indicated the relation between children with reading problems and motor control and balance. Chambers and Sugden's (246) found ineffectiveness in motor skill performance was highly associated with academic performance. Activities of daily living were found to be successfully facilitated by intervention programs that supported fine and gross motor skills thus being significant factors in early childhood learning [(247), p. 50]. The evidence indicates that physical development is fundamental to the development of a child's cognitive abilities.

Programs, based on perceptual-motor interventions have suggested that relatively simtime have not ple training is capable of moderating the facilitation of learning and brain structure (248, 249). In short, the difficulty is that the majority of current programs of this sort at the present time have not undergone rigorous evaluation and scrutiny. These types of interventional strategies invariably employ specific motor activities and exercises. Some of the advocated tasks are adjusted to the individual's needs (250), while others may be generic (251–253). These types of interventions often integrate actions, such as throwing and catching, ostensibly improving vestibular function, fine and gross motor skills, and academic accomplishment.

While some programs have promoted exercises that imitate the actions of fetuses and infants, it has been noted that rehearsing the activities of the early stages of development can inhibit the perseverance of RPRs. This has oftentimes been used as a justification for programs that advocate exercises that simulate fetal and infant activity and infants (238, 239, 254). Claims have been made that movements following primitive reflex patterns will inhibit those reflexes and improve cognitive function and the ability to acquire academic skills (239).

Grzywniak (255) studies the effectiveness of exercises aimed at supporting the development of children with learning difficulties and RPRs. Their symptoms included visual-motor coordination and attentional deficits, hyperactivity, and reduced visual and auditory analysis and synthesis.

While not many studies have thoroughly examined the effects of reducing RPRs on clinical outcomes in developmental disabilities in general and in ASD in particular, of note are Pimentel (256) and Anderson (143, 257), who discussed the connections between RPRs and developmental delays. The comparative studies scrutinized groups with developmental delays and RPRs (144, 160, 164, 238, 258). McPhillips and Mulhern (239) performed a double-blind study of the cognitive effects of reducing the presence of RPRs. A regression analysis study was conducted by de Bildt et al. (259). Melillo (260) and Hyatt et al. (252) produced meta-analyses. While at least eight systematic review articles examined, RPRs alone, or meta-analyses of RPRs in development, none explored the evidence surrounding reflex-based interventions (108, 159, 241, 252, 260–263). Expert review studies over many years included those by Rider (264), Endler (265), Ottenbacher (262), Smith et al. (266), and Mailloux et al. (267). The studies analyzed RPRs of individuals with reflex delays, developmental disabilities, and difficulties with sensory integration skills, but none provided evidence for effectiveness in the treatment of developmental disabilities of any kind by RPR reduction.

Barret et al. (268) identified three best evidence reviews (144, 238, 242). Goddard Blythe (242) provided a summary of independent studies that examined academic performance associated with developmental exercise programs that demonstrated positive effects on academic performance. Jordan-Black (238) conducted a nonrandomized control study to establish evidence for the effectiveness of reflex-based interventions in improving academic performance, from which she indicated that her system reduces RPRs in particular the asymmetrical tonic neck reflex, and has a significant effect on reading and mathematics abilities of children who had undergone her intervention. Chinello et al. (144) found that the parents of infants demonstrating subclinical traits of ASD were more likely to demonstrate RPRs and were more susceptible to developing ASD later. The study did not measure the outcome of therapeutic intervention. Collectively, these papers demonstrate the best evidence and relevance to RPR reduction and its effect on cognitive function. reflex integration. Only Goddard Blythe (242) and Chinello et al. (144), examined the effects of RPRs in developmental disorders in general and to some extent in ASD. Jordan-Black's (238) study did not address children with ASD.

Although the mechanism of how these exercises can inhibit primitive reflexes and affect or improve neurobehavioral disorders has not been previously described to our knowledge. We speculate that utilizing these exercises can increase the sensory stimulation and feedback to the nervous system that stimulates synaptogenesis and neuroplasticity of more rostral and complex areas of the brain (269–271). We conjecture that this may be associated with inhibition through descending propriospinal connections that inhibit these reflexes that would, under normal circumstances, lead to more complex individualized volitional control of movement that will stimulate growth and cortical maturity. Ultimately, this can lead to the release of “bottom-up interference” that can delay the cortex's maturation and prevent appropriate top-down regulation that will ultimately inhibit primitive reflexes (25, 27, 44, 94).

## Discussion

The incidence of ASD has been increasing at epidemic levels, and we think that the driving force behind this increase is a combination of genetic and environmental factors, emphasizing environmental determinants. We think that epigenetic factors related to lifestyle changes over the past two decades, especially reduction of early motor activity and spatial exploration of children, have led, in part, to the significant rise in the incidence of ASD. We surmise that the reduction in the activation of activity and experience-dependent genes that stimulate synaptogenesis and neuronal plasticity of central neurons and glial cells can help increase the size and complexity of the brain during the first 3 years of life. We think that this is the basis of both the maturational cortical delay identified in almost all neurobehavioral disorders, including ASD, and their associated RPRs (272–276). After the first few months of life, the feedback created by primitive reflex-generated movement can lead ultimately to the inhibition of these reflexes and the activation of more complex subsequent postural reflexes (64, 211, 277) resulting in a more complex interaction with the environment, that in turn leads to greater sensory feedback thereby activating genes that allow for the creation of integration and coordination between various cortical networks [for a more detailed analysis cf. (44)]. As these cortical networks become more connected and integrated, they increase the speed of their interaction, and their synchronization improves, allowing more areas to be activated simultaneously (24, 39, 278–282).

Delayed cortical maturity and motor coordination may occur due to the abnormal persistence of primitive reflexes. In that case, the brain will not continue to grow and develop at a normal rate and sequence. As the brain's hemispheres develop at different rates and times (283, 284), with the abnormal, asymmetric persistence of primitive reflexes, a maturational dysfunction in between hemisphere coherence can be produced where one hemisphere may mature at an average rate and the other not (231, 285–288). This can be associated with significant synchronization and temporal coherence dysfunction, decreasing large cortical networks between the two hemispheres from binding temporally and spatially. This can result in a functional disconnection syndrome (88, 102, 135, 137, 279, 289, 290).

We can conclude based on our current understanding that if there is any delay in maturation of the pyramidal tracts, the brainstem, or the frontal lobe, it is reasonable to assume that there might be a delay in the disappearance of the Babinski sign and other primitive reflexes. Many neurologists and pediatricians assume that the Babinski sign and other primitive reflexes that are present at birth and sustained for the first year of life will automatically disappear after that initial period unless brain damage is present (25, 27, 40, 94, 95, 233, 291).

Therefore, primitive reflexes are the tools that can be employed early in neonatal and infant development to evaluate the integrity of the central nervous system. They are brainstem-mediated, automatic movement patterns present in full-term infants at birth. With the maturation of the central nervous system, these primitive reflexes become more challenging to evoke after the first year postpartum when the infant becomes capable of voluntary motor activity. RPRs are not infrequently present in children with ASD, ADHD, or cerebral palsy, and may be early indicators of brain-based deficits (3, 4, 22, 148, 292–295).

## Conclusions

Retained primitive reflexes may occur in the absence of any injury. They could be used as a clinical sign of a maturational delay in cortical development that is thought by many to be highly associated with abnormal functional connectivity seen in many neurobehavioral disorders such as ASD. We have also demonstrated that the Babinski sign may reappear later in life is grossly intact in older individuals without any physical damage but is highly related to cognitive decline. This may be a functional degeneration or loss of frontal lobe function, and we can observe this with “frontal release signs.” Therefore, the presence of the Babinski's sign in a healthy aged population may be a “returned” reflex which may be an early clinical sign of frontal lobe dysfunction or degeneration, or it may represent that the Babinski reflex has been present in an individual throughout his or her lifetime. Even though they may be grossly healthy, these individuals may have struggled with neurobehavioral symptoms. This maturation process commences from the bottom of the brainstem and the primitive reflexes. They help promote bottom-up development, which then promotes growth and maturation of the brain and frontal lobes, which then, in top-down feedback, regulates the brainstem nuclei, coherent with Huglings Jackson, the Principles of Dissolution.

In conclusion, we hypothesize that RPRs in ASD are, in part, associated with maturational delays and imbalances and not necessarily a result of actual structural damage or pathology. They are, in part, a result of environmental influences and are therefore amenable to remediation. We think that the presence of RPRs and the developmental milestones that might be delayed or absent as a result may be the earliest markers of developmentally delayed children, in general, and those with ASDs in particular. Assessing RPRs in ASD then is essential for multiple reasons. Firstly, RPRs may be a possible biomarker for ASD that, jointly with early signs of attentional deficit, eye contact, and other factors, might aid in characterizing the developmental trajectory of the character of ASD in infancy. In line with this approach, it has been stated that slight disparities in motor behavior in early development (i.e., RPRs) might exert an adverse cascading effect on the subsequent development of motor skills and also in numerous other domains (i.e., communication and social behavior and /or object exploration). Secondly, the detection of early motor abnormalities could also be an encouraging avenue for the delineation of subtypes of ASD.

## Author Contributions

GL, RM, and CM: conceptualization and methodology. SK: software, visualization, validation, and investigation. GL, RM, CM, YM-F, MC-A, and TM: writing—original draft preparation. GL and EC*:* supervision. GL, RM, CM, YM-F, MC-A, TM, and EC: writing—reviewing and editing. All authors contributed to the article and approved the submitted version.

## Funding

This work was funded by a grant from the National Institute for Brain and Rehabilitation Sciences to GL and EC.

## Conflict of Interest

The authors declare that the research was conducted in the absence of any commercial or financial relationships that could be construed as a potential conflict of interest.

## Publisher's Note

All claims expressed in this article are solely those of the authors and do not necessarily represent those of their affiliated organizations, or those of the publisher, the editors and the reviewers. Any product that may be evaluated in this article, or claim that may be made by its manufacturer, is not guaranteed or endorsed by the publisher.
